# “It’s ok that I feel like this”: a qualitative study of adolescents’ and parents’ experiences of facilitators, mechanisms of change and outcomes in a joint emotion regulation group skills training

**DOI:** 10.1186/s12888-023-05080-5

**Published:** 2023-08-15

**Authors:** K Holmqvist Larsson, M Thunberg, A-C Münger, G Andersson, F Falkenström, M Zetterqvist

**Affiliations:** 1 Department of Child and Adolescent Psychiatry in Linköping, Region Östergötland, and Center for Social and Affective Neuroscience, Department of Biomedical and Clinical Sciences, Linköping University, Linköping, Sweden; 2 Department of Child and Adolescent Psychiatry in Norrköping, Region Östergötland, Norrköping, Sweden; 3 Barnafrid at Department of Biomedical and Clinical Sciences, Swedish National Center on Violence Against Children, Linköping University, Linköping University, Linköping, Sweden; 4 Department of Behavioural Sciences and Learning, Department of Biomedical and Clinical Sciences, Linköping University, Linköping, Sweden; 5 Department of Clinical Neuroscience, Karolinska Institute, Stockholm, Sweden; 6 Department of Psychology, Linnaeus University, Växjö, Sweden; 7 Department of Behavioural Sciences and Learning, Linköping University, Linköping, Sweden

**Keywords:** Emotion regulation, Skills training, Adolescents, Outcomes, Mechanisms of change, Facilitators

## Abstract

**Background:**

Emotion regulation difficulties underlie several psychiatric conditions, and treatments that focus on improving emotion regulation can have an effect on a broad range of symptoms. However, participants’ in-depth experiences of participating in emotion regulation treatments have not been much studied. In this qualitative study, we investigated participants’ experiences of a joint emotion regulation group skills training in a child and adolescent psychiatric outpatient setting.

**Methods:**

Twenty-one participants (10 adolescents and 11 parents) were interviewed about their experiences after they had participated in a seven-session transdiagnostic emotion regulation skills training for adolescents and parents. The aim of the skills training was to decrease emotion regulation difficulties, increase emotional awareness, reduce psychiatric symptoms, and enhance quality of life. The skills training consisted of psychoeducation about emotions and skills for regulating emotions. The interviews were transcribed and analysed using reflexive thematic analysis.

**Results:**

The analysis resulted in three overarching themes: Parent – Child processes, Individual processes, and Group processes. The result showed that participants considered an improved parent-child relationship to be the main outcome. Increased knowledge, emotion regulation skills and behavioural change were conceptualised as both mechanisms of change and outcomes. The group format, and the fact that parents and adolescents participated together, were seen as facilitators. Furthermore, the participants experienced targeting emotions in skills training as meaningful and helpful.

**Conclusion:**

The results highlight the potential benefits of providing emotion regulation skills training for adolescents and parents together in a group format to improve the parent-child relationship and enable the opportunity to learn skills.

## Background

Being human means having to deal with both positive and negative emotions, in ourselves and in others. Successful strategies for regulating emotions are often related to well-being, whereas difficulties within this domain tend to be associated with negative outcomes. Emotion regulation (ER) difficulties can be an underlying factor in many psychiatric conditions [1–3]. The definition of ER concerns the strategies used to influence the intensity, duration, and expression of emotions [4, 5]. Lack of adaptive emotion regulation strategies can lead to dysregulated emotions, namely an emotional experience or expression that interferes with appropriate goal-directed activity [3, 4, 6]. In children and adolescents, difficulties with ER and the use of maladaptive strategies to regulate emotions, increase the risk of developing externalising and internalising symptoms [7]. Increased symptomatology, in turn, typically contributes to increased difficulties regulating emotions [2, 8, 9]. In a meta-analysis, Compas et al. [10] examined the relationship between internalising and externalising symptoms of psychopathology in childhood and adolescence and different emotion regulation strategies. Overall, emotion regulation was significantly negatively associated with both internalising and externalising symptoms. Greater use of emotion regulation strategies was associated with lower levels of symptoms. They concluded that emotional suppression was significantly positively associated with internalising symptoms, and avoidance was significantly positively associated with both internalising and externalising symptoms. Findings from an earlier meta-analysis in the role of emotion regulation in psychopathology indicate that heightened levels of maladaptive emotion regulation strategies predict greater psychopathology, even when adaptive strategies are present [7].

The ability to regulate emotions develops throughout life [10]. In childhood, parents play an important role in teaching children how to regulate emotions [11, 12]. If parents experience difficulties in this domain, it can influence the child’s ability to develop emotion regulation strategies [13, 14]. There is some support for the observation that the child’s ability to regulate emotions improves when parents support the child’s emotional expression [11, 15].

Adolescence is a sensitive period in life with rapid development changes [16]. A maladaptive shift in emotion regulation has been seen in adolescents aged 12–15 years, compared to both younger and older adolescents [17–19]. Cracco’s [17] findings showed a reduced use of adaptive emotion regulation strategies and increased use of maladaptive strategies in adolescents during this age period. Maladaptive emotion regulation seems to be associated with a of risk of developing psychopathology [20] and psychiatric disorders often have their onset during adolescence [21]. Taken together, this points to a need of targeting emotion regulation in psychological treatments during adolescence.

Strategies to regulate emotions can be acquired through treatment, such as skills training. Research has shown that emotion regulation is one important mechanism underlying the effects of some psychological treatments [22]. Treatments that emphasise emotion regulation as a core component show positive results in reducing nonsuicidal self-injury (NSSI), symptoms of borderline personality disorder, and depression and anxiety [23–28]. Changes in emotion regulation has also been shown to mediate improvement in NSSI [29]. During the last decades several treatments have been developed that target emotion regulation skills. Dialectical Behaviour Therapy (DBT [30]) is one of the most disseminated of these treatments. DBT has showed promising results as a transdiagnostic treatment for depression and anxiety [31, 32], and has also been tested in randomised controlled trials for adolescents [27, 33]. Another example is Emotion Regulation Group Therapy (ERGT [34]) which is based on DBT and Acceptance and Commitment Therapy (ACT [35]) and was developed for BPD and self-harm. Positive results for ERGT have been observed in treatment studies [26, 36], with increased emotion regulation skills and overall quality of life, and reduced self-harm, symptoms of BPD and depression [37]. A further example is Barlow’s transdiagnostic treatment Unified Protocol (UP [23]), with promising effects on anxiety and depression for adults in both individual and group format [28, 38, 39]. The above-mentioned studies were all originally developed for adults but have been modified for adolescents [40–42]. Emotion Regulation Individual Therapy for Adolescents (ERITA [24, 40]), for example, has been tested as an online treatment for adolescents with nonsuicidal self-injury aged 13–17 years. Results from these studies show that ERITA is feasible and that participants decrease their nonsuicidal self-injury and emotion regulation difficulties. Difficulties with emotion regulation is typically viewed as a transdiagnostic construct. In psychological treatment, transdiagnostic approaches have the potential of being beneficial for patients with several comorbidities [43].

When developing effective treatments, it is important to examine mechanisms of change [44]. In DBT, for example, emotion regulation skills that help to downregulate emotional reactions seem to be one mechanism that influences treatment outcome [45].

In summary, maladaptive skills or lack of skills to regulate emotions can lead to frequent and problematic dysregulated emotions, which in turn can contribute to impulsive and destructive behaviours, psychiatric symptomatology, suffering and reduced quality of life [2]. On the other hand, improved emotion regulation is a mechanism of change in several psychiatric conditions [36, 45]. Based on this research, we developed a transdiagnostic emotion regulation skills training for adolescents and parents in child and adolescent psychiatry (CAP). This study is part of a randomised controlled trial that aims at exploring if a brief skills training in emotion regulation can reduce difficulties with emotion regulation in a clinical sample. The aim of the skills training was to decrease emotion regulation difficulties by learning emotion regulation skills, decrease impulsive behaviours, reduce psychiatric symptoms, and enhance quality of life [46]. Based on the research described above on the consequences of suppressing and avoiding emotions, the skills training focused on skills for becoming aware of and accepting emotions rather than controlling them, in addition to psychoeducation about emotions.

Based on earlier findings that parents play an important role in children’s socialisation of emotions and in the development of ER strategies, and the fact that there are advantages for treatment outcome when adolescents and parents can learn and practice new skills together [47], the skills training was delivered in a group format jointly with adolescents and parents. In a pilot study, the skills training was feasible and showed promising results in decreasing emotion regulation difficulties in both adolescents and parents [46].

When developing new treatments, qualitative research has the possibility of providing a richer understanding of how the intervention is perceived by participants. A qualitative analysis can provide an in-depth perspective of facilitators, mechanisms of change and outcomes in treatment. It has the potential to illustrate what participants themselves perceive as helpful and meaningful. Another advantage is that participants can reflect freely on their experiences of the treatment effect, in addition to researchers’ hypotheses.

A few qualitative studies have examined adolescents’ and caregivers’ experiences of participating in skills training groups. Participants report that a transdiagnostic approach was appreciated, focusing on emotion regulation skills was helpful, and the group format was an important factor [48–51]. A qualitative study of the online treatment Emotion Regulation Individual Therapy for Adolescents (ERITA; [24]) showed that decreased difficulties with emotion regulation was a valuable treatment outcome for adolescents [51]. Since earlier research has found that participants overall seem to appreciate skills training in a group setting [48, 49], we wanted to examine how participants experienced a joint group skills training where adolescents and parents participate together. To our knowledge, no other study has examined both adolescents’ and parents’ experiences of a joint emotion regulation skills training. Thus, this study aimed to examine participants’ experiences of participating in a joint emotion regulation group skills training in a CAP setting.

## Method

This qualitative study is part of a randomised controlled trial of an emotion regulation skills training for adolescents and parents where both quantitative and qualitative data were collected. The setting was two CAP outpatient clinics in the county of Östergötland, Sweden. The study is presented according to the Consolidated criteria for reporting qualitative research [52].

### Intervention

The skills training was developed by the first and last author informed by evidence-based CBT treatments with emotion regulation as a core component, such as DBT [30], Emotion Regulation Group Therapy [34], Acceptance and Commitment Therapy [53], Unified Protocol [23], and from clinical experiences of treating adolescents in CAP.

The overall aim of the skills training was to decrease difficulties with emotion regulation, and psychiatric symptoms, increase emotional awareness and enhance levels of functioning and quality of life. The content of the skills training focused on psychoeducation about emotions and emotion regulation skills by training participants in increased awareness and knowledge of what emotions are and how emotions work; understanding the functions of emotions; increasing skills in identifying and labelling emotions; expressing primary emotions and self-validation; regulating and accepting emotions and acting in accordance with their valued direction, even in moments of strong emotional arousal, and also reducing emotional vulnerability. See Table 1. The course format was seven two-hour, weekly sessions, and a booster session after three months. Adolescents and parents participated together. Each session followed the same structure: repeat content of previous session, go through homework assignments, have a break with snack before a new theme and skills are presented, which results in a new homework assignment.

**Table 1:** Overview of the Content of the Emotion Regulation Skills Training

Sessions	Content
Session 1	Awareness of emotions and labelling emotions
Session 2	Identifying emotions and the functions of emotions
Session 3	Primary and secondary emotion. Validating and reducing judgment
Session 4	Reducing vulnerability and emotional imbalance
Session 5	Making conscious choices – goal directed behaviours
Session 6	Acceptance and valued directions
Session 7	Repetition
Booster session	Repetition and maintenance

### Participants

The skills training was transdiagnostic and open to patients 14–17 years old. Exclusion criteria were current substance abuse, severe anorexia, schizophrenia, and ongoing psychosis. To participate in the study, participants had to attend at least one session of skills training during 2021. Selection of participants for interviews was made to maximise the variation in the sample and included both adolescents and adults in different family constellations, of different sexes and ages, from both clinics and with different skills trainers. Gender (in both adolescents and parents) and family constellation were prioritised. A total of 21 participants were interviewed. The sample consisted of 11 (seven mothers and four fathers) parents (P1-P11) and 10 (nine girls and one boy) adolescents (A1-A10). Nine adolescents and ten parents were related. In one family, two parents were interviewed. One adolescent and one parent in the sample were unrelated. The adolescents were between 14 and 18 years with several psychiatric diagnoses, most commonly depression and anxiety disorders. The participants attended four to seven sessions, M = 6.30, median = 7. See Table 2.

**Table 2:** Participants’ demographics, adolescents (n = 10) and parents (n = 11)

Variables		Frequency (%)
Adolescents		
	Females Males	9 (90.0) 1 (10.0)
	Age baseline, *m* (*sd*)	15.6 (1.36)
Parents		
	Females	7 (63.6)
	Males	4 (36.4)
DSM-5 diagnoses*^a^		
	Anxiety disorder	7 (70.0)
	Depression**	7 (70.0)
	ADHD/ADD	5 (50.0)
	Eating disorder	2 (20.0)
	Other***	4 (40.0)
Number of diagnoses^a^		
	1	1 (10.0)
	2	5 (50.0)
	3	1 (10.0)
	4	3 (30.0)
Number of sessions		
	1	0 (0.0)
	2	0 (0.0)
	3	0 (0.0)
	4	1 (10.0)
	5	1 (10.0)
	6	2 (20.0)
	7	6 (60.0)

*Note.* *DSM-5 = Diagnostic and Statistical Manual for Mental Disorders, fifth version, ADHD = Attention Deficit Hyperactivity Disorder, ADD = Attention Deficit Disorder. **Depression including persistent depressive disorder. *** For example, Oppositional Defiant Disorder, Post-traumatic stress disorder, high functioning autism. ^a^Each participant could have several diagnoses.

### Data collection

The skills trainers suggested 38 participants that met criteria for maximising the variation to the first author, who then reviewed eligible participants for variation and approached 26 participants by telephone. Of these, one did not answer and four declined participation. Due to the pandemic, 10 interviews were held digitally as video calls on Skype, 10 by telephone and one face-to-face during the period May 2021 - February 2022. Adolescents and parents were interviewed individually and separately. Interviews were conducted 3–4 months after the skills training by two female psychotherapists trained in DBT and with experience of clinical child and adolescent psychiatry (KHL and MT). Both the interviewers were emloyed at CAP-clinics. One of the interviewers (KHL) had been involved in the recruitment process to the RCT, but beyond this no one had any prior relation to the participants. The interviewers presented their occupation and role in the research project. The interview schedule was developed by three of the authors (KHL, MT and MZ) and was partly based on comments that the skills trainers had spontaneously received from earlier participants about their experience of the skills training. The interview schedule was tested on two adolescents and two parents. Nothing in the schedule was changed after the test and the four interviews were included in the analysis. The interviews were semi-structured with open questions about the experiences of participating in the skills training, participating together with a family member, the group format, and outcomes of the skills training. Examples of questions were; how did you experience participating in the emotion regulation group together with your parent/child; what did you think was the most important thing you took with you from participating in the emotion regulation group? Mean length of interviews was 20 min (varying between 8 and 38 min). Interviews were recorded and transcribed verbatim.

### Analysis

Data were analysed with reflexive thematic analysis (TA) according to Braun and Clarke [54]. The analysis was made in the six steps recommended in reflexive TA [55]. The interviews were read several times to become familiar with the material. Codes were produced individually by KHL, MT, A-CM and MZ, and themes were created and discussed together. The themes were reviewed several times and the analysis had a constant movement back and forth in the six steps. KHL and MZ reviewed different ways of clustering the codes several times until consensus was reached on which themes best covered the interpretation of the data. The final themes were reviewed by A-CM. KHL and MZ labelled the themes, and the results were discussed and approved by all authors. The research group consisted of CAP-clinicians, professors, and associated professors, from both the Faculty of Medicine and the Faculty of Philosophy. In combination, the researchers have extensive experience of quantitative and qualitative research. Both females and males were represented in the group.

## Results

In the analysis, three overarching themes were developed: Parent – child processes, Individual processes, and Group processes. See Table 3.

**Table 3:** Themes

Overarching themes	Themes
Parent – Child processes	Emotional communication
	Connectedness
	Participating together
Individual processes	Behavioural change
	Emotion regulation skills
	Knowledge
Group processes	Shared experience
	Receiving help and support
	Group climate

### Parent – child processes

**Fig. 1:**
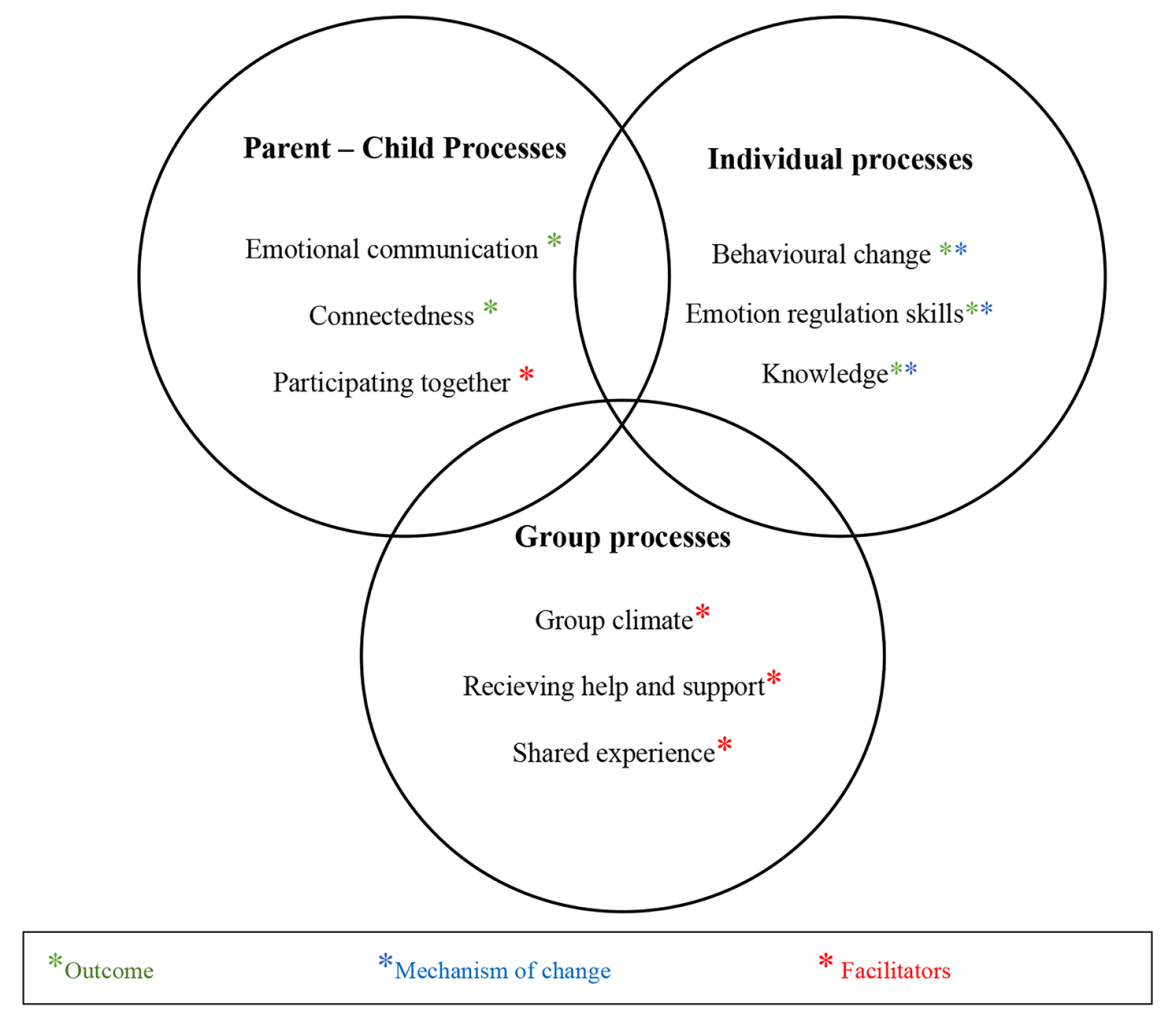
Participants’ experiences of outcomes, facilitators and mechanisms of change of a joint emotion regulation skills training for adolescents and parents

The participants described that the joint skills training affected the parent-child relationship, which was viewed as one of the most valuable outcomes. Despite the fact that the sampling process was made to maximise the variation, the result did not differ in any substantial and meaningful way between adolescents and parents, different genders or family constellations. This is in itself an interesting result, i.e., that adolescents and parents shared most experiences. We have therefore chosen to report parents’ and adolescents’ experiences together throughout the result section. In the analysis, the themes “Emotional communication” and “Connectedness” were interpreted as outcomes and the theme “Participating together” was categorised as a facilitator. See Fig. 1.

**Emotional communication.** A primary outcome that adolescents and parents described was a positive change in their communication with changes in frequency, quality, and content. Participants talked more about emotions with each other and how they felt. One adolescent said: *“I was like not saying and not showing my feelings, like, and then I think it was good anyway to be able to talk a bit about feelings with Mum, like. So that I can begin to do that more”* (A1). Adolescents described that their parents had changed their behaviour, which, in turn, had affected the adolescents’ willingness to share emotional experiences with their parents. This was expressed by one of the adolescents: *“And it has been a help because it’s not much fun to be met with anger, when you are talking about something that perhaps is tough or so. Er… so it’s helped a lot as well with communication generally and it feels easier to talk”* (A7). The skills identifying emotions, differentiating between primary and secondary emotion, validation and taking a pause appeared to be especially important for this outcome. One potential explanation behind this change in communication could be that the act of participating together in the skills training facilitated learning emotion regulation skills, such as labelling emotions, and thus participants acquired a common language for communicating about emotions.One parent noted: *“And then we could like start out from that when we wanted to move forward at home (…), because we had like the same language, the same words”* (P1).

**Connectedness.** Participants expressed increased closeness and improved parent-child relationship as outcomes of the skills training. Both parents and adolescents described that they gained new perspectives of each other, which was beneficial for the relationship. Adolescents described that the parent understood them better: *“We had a very good relationship previously as well, but now it has become a more all-round relationship as well. With a bit more understanding, perhaps”* (A7). This was conceptualised as “Connectedness” and both parents and adolescents emphasised this as an important outcome. From the parents’ perspective, this seemed to be related to a change in how much the child let the parent help them in challenging situations. One parent noted: *“She had a row with her boyfriend the other day and was upset and then I could actually go in and hug her and like… that she talks to me about it and so on”* (P2). The increased willingness of adolescents to receive help and parents’ increased opportunity to comfort, seemed to contribute to the feeling of connectedness.

**Participating together.** Parents and adolescents expressed that it was valuable to participate together in skills training. It was interesting to hear one’s own family member talk about thoughts, emotions, and difficulties in regulating emotions. *“Just getting to listen to each other and getting to listen to your own child, in fact, who is talking and thinking aloud, and that you are like sharing the same knowledge in some way, too”* (P3). Some of the adolescents described that it was in the skills training that they, for the first time, realised that parents also struggled with emotions. They described this as something positive, which contributed to a stronger connection to their parent. As one adolescent said: *“I think they [parents] need it just as much as I do, when it comes down to this in particular”* (A7). Spending time together with a parent in skills training was described as positive and something that contributed to an improved relationship. It seemed to be an important signal to the adolescent that the parent prioritised participating in the skills training. For some adolescents, participating together with a parent was a prerequisite for being able to participate in the skills training. Several adolescents described that they felt safe having their parents in the group, *“It felt quite secure because I had my Mum there. (…) I don’t know, it was just a sense of security”* (A6). A few adolescents also expressed that it would have been beneficial if one of the sessions was separate, as it would have been easier to talk about certain things without the parents.

### Individual processes

The overarching theme “Individual processes” contains different types of intrapersonal processes that were developed in the analysis. Participants described the importance of knowledge, what they had learnt, which skills were valuable and useful, and what behavioural changes they had made. The skills training consisted of two parts, psychoeducation, and learning new skills, which was reflected in the themes. The themes “Knowledge”, “ER skills” and “Behavioural change” were interpreted as both mechanisms of change and outcomes. See Fig. 1.

**Knowledge.** Participants described that a significant component of the skills training was the knowledge they gained about emotions and their functions. Several participants described the importance of understanding *why* things happen. In the analysis, “Knowledge” was interpreted as both a mechanism of change and outcome, as participants described that it was easier to change their behaviour and regulate emotions based on this new knowledge, but also that increased knowledge was an outcome. One adolescent expressed that increased knowledge in itself was helpful, *“You don’t have to be scared of it, it’s my brain that has fooled me into being scared of it”*(A3). Knowledge was also something the parents commonly referred to, and several parents questioned why they had not been introduced to this information earlier in the child’s life. One parent noted: *“There were several reflections in the group that this is, shit, this is actually something that everyone should know”* (P4).

**ER Skills.** Almost all skills that were taught were mentioned by the participants, but some more frequently than others. Overall, validation (self-validation and validation of others) and the ability to differentiate between primary and secondary emotions were most commonly referred to. One adolescent said: *“The most important thing that I will take with me at any rate is this thing about identifying if you are sad or angry or (…) that you pause and think about it when you are absolutely furious and just, wait a minute, am I actually sad?”* (A5). In this excerpt the adolescent described several skills: pause, identify emotion, label emotion, and differentiate between primary and secondary emotion. One parent noted: *“The absolutely most important thing (…) this thing with primary and secondary emotions, that you have to take care of the needs of the primary emotion”* (P2). This parent also addressed the importance of distinguishing between primary and secondary emotion but added an additional skill: taking care of the need embedded in the primary emotion.

**Behavioural Change.** Only a few participants reported reduced symptoms as an outcome, but several examples of concrete behaviour changes appeared. One adolescent expressed: *“Instead of feeling ‘Wow, now I must stop this because it’s stupid’, I think ‘It’s okay that I feel like this’, and then my body calms down”* (A11). These behavioural changes were interpreted as a treatment outcome. Both parents and adolescents described their own behavioural changes, but to a greater extent they described behavioural changes they had witnessed in their family member. An example can be found in this excerpt where a mother describes her daughter’s dialogue with her boyfriend: *“Even when I get angry because I have anxiety then I am actually sad, like, so…. And then it’s important that you give me a hug or something like that, that you don’t get angry as well, because that’s not what I need”* (P2). The behavioural changes also had an impact on the parent-child relationship. Using skills and trying new behaviours potentially led to positive circles of change in the dyad. One parent noted *“She felt like it was really annoying that I problem solved. I never asked how she felt or asked why, or if she wanted to talk about it, if she needed anything, if I could do anything for her. Instead I just went straight to problem-solving, and she also feels that I have got much better at that”* (P5).

### Group processes

The overarching theme “Group processes” describes the group format as a facilitator for learning and practicing new skills. See Fig. 1. The experience of not being alone, but rather sharing the same difficulties and being helped by other participants, were aspects that participants emphasised. In particular, the validating experience of not being the only one having difficulties with emotions was something that seemed important. Overall, participants were positive to the group format and described the positive effects of getting skills training in group.

**Shared experience.** The experience of being part of a group was something the participants addressed as helpful. A positive aspect of taking part of others’ narratives was a sense of not being alone. One adolescent expressed: *“That like takes away the ’I’m alone in the world’ feeling”* (A3). Parents also described the validating experience of sharing emotional challenges with others as crucial for being able to share own experiences: *“You noticed that you were struggling with the same things”* (P6).

**Receiving help and support.** Participants described receiving help and support, not only from the skills trainers, but also from group members. Help could come in the form of ideas, tips or being validated from others, *“It’s good to hear (.) what other people do and what other people think and what others feel. So perhaps you can get a few ideas”* (A8). Support from the group included other group members asking questions which oneself did not dare or wasn’t able to ask. As one parent put it: *“But also that some people could put things into words that you yourself couldn’t put into words…”* (P2).

**Group Climate.** The group climate was interpreted as a facilitator for learning and practicing skills. Participants described the climate as warm and including, which contributed to a sense of security. One adolescent described: *”It wasn’t like you were embarrassed to say anything but rather that everyone was very understanding”* (A1). One aspect of the group climate, that was frequently commented on, was that the warm and nonjudgmental climate was crucial for being able to share experiences. One parent said: *“It was really good, we could even laugh at each other”* (P7).

## Discussion

In this study, adolescents’ and their parents’ experiences of facilitators, mechanisms of change and outcomes of a joint emotion regulation group skills training in a CAP outpatient setting were studied. The main finding was that improved parent-child relationships appeared to be the primary outcome of the skills training. Increased knowledge, ER skills and behavioural change were conceptualised as both outcomes and mechanisms of change, based on participants’ descriptions. The group format, and the fact that the skills training was delivered jointly, were seen as facilitators and participants appreciated the support from others in the group. Furthermore, the participants experienced targeting emotions in skills training as meaningful and helpful. Interestingly, there was a high consensus concerning reported experiences between adolescents and parents, different genders and family constellations.

### Parent-child processes

Both adolescents and parents described that the skills training affected their relationship in a positive way. The impact of the skills training on the parent-child relationship emerged as the primary outcome. This is an interesting finding, since the skills training did not primarily aim at improving family relationships, but rather targeted emotion regulation skills. Acquiring a shared language about emotions, potentially opened up a more adaptive emotional communication that brought parents and adolescents closer together. Participants in an earlier qualitative study of ERITA, in which emotion regulation skills were taught to adolescents and parents online and separately, described several positive outcomes related to increased skills but did not emphasise the parent-child relationship and improved communication between adolescent and parents to the same extent [51]. It thus seems that delivering emotion regulation skills training in a joint format has the potential to impact the parent-child relationship. Earlier research has shown that improved family relationships and communication are mechanisms of change in the treatment of adolescent externalising behaviour [56], and it would be interesting to further investigate the relationship between family functioning and emotion regulation skills.

During adolescence, children tend to strive for autonomy and less parental involvement [57]. Despite this process, several adolescents in the study described that they appreciated their parents’ presence, and, in some cases, parents’ involvement was necessary for adolescents to participate. Parents play a significant role in the development of children’s emotion regulation strategies [9, 11, 15, 58]. In a joint skills training, parents can become role models for emotion socialisation and adaptive emotion regulation. These findings have implications for the development of clinical treatments, as it is common practice to deliver skills training separately to adolescents and parents [50, 51].

A few adolescents, however, also wished to have one separate session to address topics that were difficult to talk about in the presence of parents, which is in line with Flynn’s [48] qualitative study of adolescents experience of group treatment, and is important to consider as a further development of the treatment model.

### Individual processes

The analysis showed that participants considered increased emotion regulation skills and increased knowledge as outcomes of the skills training. In accordance with earlier treatment research that focused on skills in general, and emotion regulation skills in particular, participants in the current study experienced the emotion regulation skills as helpful [48, 51, 59].

When asked specifically about this, only a few participants reported reduced psychiatric symptoms as an outcome, which contradicts earlier quantitative research where emotion regulation has been identified as a mechanism of change for symptom outcome, such as reduced NSSI [22, 36, 45]. On the other hand, participants described several behavioural changes as outcomes, such as increased validation of self and others. An interpretation of this result is that increased knowledge, and ER skills, are outcomes and mechanisms of change for individual behavioural change and for improved parent-child relationship. The psychiatric symptomatology of patients in CAP with several comorbidities, is perhaps not easily reduced after a 7-session treatment, while other behavioural outcomes related to emotion regulation are potentially more apparent for the participants.

### Group processes

The group format provides group members with a potential opportunity to practice skills in a safe environment in a context that resembles real life, and support from group members can improve motivation and treatment compliance [60]. Our results indicate that the group format itself was one potential facilitator for behavioural change. Both adolescents and parents mentioned that it was validating to hear other participants describe their struggles with emotion dysregulation, which was important for being able to share their own experiences. This is in line with previous research showing that group functioning and bonding with group members and leaders, were associated with reduced anxiety, aggression and social competence [61]. Results from a qualitative study of parents in a skills training group indicated that being in a group where suffering and emotional experiences were shared, opened up the possibility to receive support and to learn from other participants [48].

Our results also showed that the group format could reduce participants’ feelings of being alone. Since loneliness and social isolation are known risk factors for mental health problems [62], skills training in group could be recommended to patients to reduce feelings of loneliness.

### Limitations

The results need to be interpreted in light of some limitations. There could be a potential bias in those that chose to participate in the skills training. Families with severe interpersonal conflicts or problems were perhaps not as likely to participate in a joint skills training. There could also be a bias in which adolescents would consider participating in a group intervention. Perceptions of the skills training could potentially differ depending on diagnoses, number of sessions attended and the adjacent ongoing treatment in the CAP setting. Further research is needed to confirm the results.

Some of the interviews were relatively short, which could potentially affect the result. The format (telephone and digital video meetings) may have impacted the length of the interviews, if some participants found it easier to communicate face-to-face. Longer in-depth interviews made face-to-face could possibly had given fuller descriptions from the participants.

Another limitation is the potential bias of the analysis team. Three of the authors that took part in the analysis have a background in CAP and were trained in treatments that focus on emotion regulation skills. Furthermore, two of the authors were the developers of the skills training. This could possibly have contributed to a more positive interpretation of the data. In an attempt to compensate for this potential bias and validate the themes, one of the members in the reflexive analysis team did not have a background in CAP or emotion regulation treatments, and instead had extensive experience in qualitative research. Further research is thus needed on other patient samples, in other settings, and by other researchers to confirm the results.

## Conclusions

Understanding why and how treatment works are important challenges in psychotherapy research [63]. This study contributes important knowledge on participants’ experiences of these processes. The study highlights adolescents’ and parents’ experiences of facilitators, mechanisms of change, and outcomes of a joint emotion regulation skills training in a clinical setting. The group format, and the fact that the skills training was delivered jointly were appreciated and potential facilitators for learning skills. Increased knowledge, increased ER skills and behavioural change were interpreted as both mechanisms of change and outcomes, and improved parent-child relationship was identified as an outcome.

## Data Availability

The data that support the findings of this study cannot be made publicly available for confidentiality reasons. Data are however available from the corresponding author upon reasonable request subject to ethical permissions and participant consent.
